# Investigating Students’ Perceptions towards Artificial Intelligence in Medical Education

**DOI:** 10.3390/healthcare11091298

**Published:** 2023-05-01

**Authors:** Ali Jasem Buabbas, Brouj Miskin, Amar Ali Alnaqi, Adel K. Ayed, Abrar Abdulmohsen Shehab, Shabbir Syed-Abdul, Mohy Uddin

**Affiliations:** 1 Department of Community Medicine and Behavioral Sciences, Faculty of Medicine, Kuwait University, Jabriya 046300, Kuwait; 2 Ministry of Health, Jamal Abdel Nasser Street, Sulaibkhat, Kuwait City 13001, Kuwait; b.miskin@moh.gov.kw; 3 Department of Surgery, Faculty of Medicine, Kuwait University, Jabriya 046300, Kuwait; amar.alnaqi@hsc.edu.kw (A.A.A.); adel@hsc.edu.kw (A.K.A.); 4 Department of Immunology, Mubarak Alkabeer Hospital, Hawalli Health Region, Ministry of Health, Jabriya 047060, Kuwait; abrar.shehab2@mail.dcu.ie; 5 Graduate Institute of Bioinformatics, School of Gerontology and Long-Term Care, Taipei Medical University, Taipei 100-116, Taiwan; drshabbir@tmu.edu.tw; 6 International Center for Health Information Technology, Taipei Medical University, Taipei 100-116, Taiwan; 7 Research Quality Management Section, King Abdullah International Medical Research Center, King Saud bin Abdulaziz University for Health Sciences, Ministry of National Guard-Health Affairs, Riyadh 11481, Saudi Arabia; drmohyuddin@yahoo.com

**Keywords:** artificial intelligence, medical education, medical students, perception, understanding, awareness

## Abstract

Implementing a reform in medical education requires students’ awareness regarding the importance of artificial intelligence (AI) in modern medical practice. The objective of this study was to investigate students’ perceptions of AI in medical education. A cross-sectional survey was conducted from June 2021 to November 2021 using an online questionnaire to collect data from medical students in the Faculty of Medicine at Kuwait University, Kuwait. The response rate for the survey was 51%, with a sample size of 352. Most students (349 (99.1%)) agreed that AI would play an important role in healthcare. More than half of the students (213 (60.5%)) understood the basic principles of AI, and (329 (93.4%)) students showed comfort with AI terminology. Many students (329 (83.5%)) believed that learning about AI would benefit their careers, and (289 (82.1%)) believed that medical students should receive AI teaching or training. The study revealed that most students had positive perceptions of AI. Undoubtedly, the role of AI in the future of medicine will be significant, and AI-based medical practice is required. There was a strong consensus that AI will not replace doctors but will drastically transform healthcare practices.

## 1. Introduction

Artificial Intelligence (AI) simply means making machines capable of simulating intelligence by giving the computer human-like capabilities, such as understanding, reasoning and problem-solving. AI interprets external data, learns from it, and uses this learning to achieve specific goals and tasks [1]. In the medical context, the applications of AI are aimed at “supporting decision-based medical tasks through knowledge- and/or data-intensive computer-based solutions that ultimately support and improve the performance of a human care provider” [2]. AI systems can perform numerous functions to provide support to clinicians in various medical fields, such as drug development, disease diagnostics; health monitoring, medical data management, personalized medicine; and in the analysis of health plans, surgical treatments, and medical treatments [3]. Lately, AI systems have gained popularity in the field of medicine after successful implementations of AI-based Machine Learning (ML) algorithms.

Introducing AI applications in healthcare can transform the way that clinicians practice medicine by increasing diagnostic accuracy and the appropriateness of treatments. Such reforms require clinicians to have a good understanding and knowledge of AI and a willingness to use AI applications efficiently [3,4,5]. In the literature, many studies have been reported on the use of AI applications in diverse medical specialties, most dominantly in the diagnostic radiology [4,6], pathology [7], cardiovascular medicine [8], ophthalmology [9], dentistry [3], and dermatology [10].

Two forms of AI hold great importance. Firstly, ML, which is a subset of AI that uses algorithms programmed on multiple examples and datasets to identify patterns in order to make machines “learn” to perform specific tasks and intelligent predictions [11,12]. Several applications of this technique can be seen in various fields. For example, in engineering, the artificial neural networks technique was used to design bridge-type compliant mechanism flexure hinges with optimal dimensions [13]. Another example can be seen in an improved design of a centrifugal pump with the help of AI algorithms [14]. While in medicine, machine learning is very promising; it allows the insights gleaned from the choices made by countless clinicians and the results obtained from billions of patients to guide the treatment of each individual patient, considering at the same time each patient’s unique circumstances. This can be seen in existing models [15], which include: (a) models to identify hospitalized patients with poor prognosis and risk of transfer to intensive care units; (b) models to identify abnormalities on medical images to aid in diagnosis; and (c) models to automatically select patients who might be eligible for randomized controlled trials, based on their clinical records. However, some challenges face machine learning use in medicine, such as the need for high-quality data to train the models, the possibility of ML using undesirable past practices, and the demand for expertise to oversee, regulate, and set the limitations [15].

The second form is the large language models (LLMs). Recent advances in LLMs have the potential to reshape various aspects of medical practice, as well as education, including knowledge retrieval, clinical decision support, and summarization of key findings. Using evaluation frameworks is, however, necessary to measure the progress and mitigate the harms [16]. One application of LLMs that has been used with increasing frequency is chatbots. Chatbots consist of an AI system and a chat interface and are operated using a “prompt”, meaning a request or query, to which the chatbot then gives a natural language “response”. ChatGPT (Generative Pretrained Transformer) is a general AI chatbot developed by OpenAI company and is recently being widely used for all kinds of purposes. GPT-4 is the most recent version of ChatGPT that has been publicly released as of March 2023. Some possible applications of these technologies include, but are not limited to, medical note writing, suggesting further workup and prescription orders, answering questions about innate medical knowledge, providing consultation services, providing information for patients, and utilization in scientific writing and healthcare research [17,18]. However, chatbots, such as GPT-4, as well as other similar AI systems, are still a work in progress and have important limitations, which include the possibility of making mistakes [17]. Moreover, ethical issues, bias, and limited knowledge are some concerns regarding ChatGPT [18]. Certainly, these applications are the subject of debate and discussion in the medical and public community [17].

Thus, attention has to be paid to the medical education of the next generation of doctors, as they need to be well-equipped with advanced clinical practices, which can be performed via AI applications. These applications require new competencies for utilizing a massive medical dataset and analyzing and/or forecasting outcomes. Thus, future clinicians should become educated users to objectively analyze the use of AI systems, better understand AI concepts, and assess discrepancies between algorithms generated for medical tasks [19].

There is enough literature on the knowledge, attitude, and perceptions of healthcare professionals and medical students toward the adoption of AI and ML in clinical practices and medical education, respectively. This includes studies that reflect both sides, i.e., awareness about AI as well as lack of AI knowledge in clinicians and students. For example, a Korean study explored doctors’ attitudes towards the adoption of AI systems in healthcare and concluded that most of them were not familiar with AI but agreed on the usefulness of AI in medical practices; moreover, the doctors also stated that AI could not replace their roles [20]. On the other hand, misunderstandings about AI can create fear among users about adopting it in the future. These outcomes were reported in a Swiss study that found a lack of AI knowledge among a small batch of medical students (n = 55), which was considered to be a potential threat to the application of AI in the diagnostic radiology [21,22]. Therefore, it is imperative to avoid confusion concerning the concept of AI in medicine and improve awareness early on. Overall, medical education should not only teach the foundation of biomedical and clinical sciences but should also cover the broad range of skills that are required for future physicians to be effective in their use of AI systems in clinical practices, especially as the adoption of AI continues to grow in healthcare [22,23,24].

In 2007, the Association of American Medical Colleges (AAMC) recommended that medical schools should incorporate technology into medical education, such as the use of simulation technologies [25]. With the emergence of AI-based ML, medical education needs to move from the information age to the AI age to support clinical practice with appropriate decision-support [23]. Despite this, due to the lack of systematic evidence, the incorporation of AI into undergraduate medical education remains limited [26].

Incorporating AI concepts and applications into the medical curriculum can be advantageous for students, wherein AI systems can play an important role in the education process [22]. These systems can provide the following features: (1) a multi-sensory and stimulating environment, which can increase students’ attention and participation in the learning process so that knowledge acquisition will be improved and competencies will be gained, (2) feedback about learners’ performance, which can help in monitoring their level of improvement, (3) opportunities to engage in problem-based learning at any time and with a variety of cases, without danger to real patients, and (4) an opportunity to observe medical cases on a model in a variety of grades, introducing different interventions in a safe environment.

Implementing a technological reform in medical education requires students’ readiness and awareness regarding the importance of adopting the latest technologies, such as AI concepts, in order to equip themselves with the knowledge and skills required for future medical practice.

Within the Arab region, only a few recent studies have assessed the medical students’ knowledge of AI; for example, a Jordanian study investigated knowledge of AI and ML among medical students from six universities in Jordan [27]; an Omani study examined the impact of AI on social and computer anxiety in e-learning settings for students at Al Buraimi University College in Oman [28]; and another Saudi study that looked at teachers’ and students’ perceptions for the impact of AI on English language learning in Yanbu University [29]. Interestingly, a systematic review was recently conducted that comprehensively analyzed the role of AI in education in general for the Arab world in the past five years [30]. Our study aimed specifically to investigate students’ perceptions of adopting AI systems in medical education and their readiness to adopt AI systems in their learning within Kuwait University’s Faculty of Medicine (KUFoM), Kuwait. To the best of our knowledge, no research of this kind has been performed in any of the academic institutions of Kuwait to date.

## 2. Methods

### 2.1. Study Design, Population, and Research Setting

A cross-sectional survey study was conducted using an online questionnaire to collect data from students of KUFoM from June 2021 until November 2021. The undergraduate medical program at KUFoM involves three phases, namely: a foundation year (phase I), a pre-clinical phase (phase II), which takes three years of duration, and a clinical phase (phase III), which also takes three years. This study was only conducted with students of phase II and phase III and excluded the foundation year, wherein basic sciences are taught. Three reminders were sent out to the respondents.

### 2.2. The Survey Questionnaire

The items of the questionnaire were taken from two previous validated studies [5,31]. In order to ensure the contents validity of the questionnaire items and to match the objectives of this study, the questionnaire was revised by an academic team that consisted of three assistant professors from the faculty of medicine and a senior lecturer with a health informatics background from faculty of allied health sciences at Kuwait University. In this process, the items were reviewed for the clarity of wording to avoid ambiguous statements and customized to ensure that they were free from wording bias. Additionally, it was assured that the scale used was appropriate and compatible with the items’ wordings. The pilot study was conducted with 20 students to examine the suitability and readability of the questionnaire, and no specific feedback for any required modification was reported. The reliability of the questionnaire items was measured using the test-retest technique, where the questionnaire was given twice to the same students at different times. Twenty students were tested on Saturday and then retested the following Saturday. Accordingly, the total scores of the two tests were correlated, and the resulting intraclass correlation value (0.984) indicated a very high correlation (excellent reliability).

The questionnaire consisted of three sections, namely (1) demographic data, (2) perceptions of AI, and (3) the impact of AI on medical education.

The questionnaire was developed in an electronic format using online Google Forms. The data collection was achieved by distributing the link to the questionnaire via the university email system to all medical students in phases II and III.

### 2.3. Ethical Consideration

Ethical approval for the study was obtained from the Health Sciences Centre Ethical Committee at Kuwait University (Reference number: VDR/EC/3730). The study was conducted in accordance with the principles and guidelines of the Declaration of Helsinki for medical research involving human subjects [32]. Informed consent was obtained from all participants who agreed to participate in completing the questionnaire for this study.

### 2.4. Construct Validity of the Questionnaire’s Items

The construct validity of the two scales in the questionnaire was tested using factor analysis.

#### 2.4.1. Students’ Perceptions Scale (10 Items)

A factor analysis test was performed on ten items; one common factor was extracted for nine items, and one item (item no. 2) looked weak (less than 0.30) (see Table 1). The reliability test on the ten items indicated an acceptable level, where Cronbach’s Alpha value was 0.755.

#### 2.4.2. Impact of AI on Medical Education (Five Items)

A factor analysis test was performed on five items; one common factor was extracted for four items, and one item (item no. 4) looked weak (less than 0.30) (see Table 2). The reliability test on the five items indicated an acceptable level of reliability, where Cronbach’s Alpha value was 0.635.

### 2.5. Statistical Analysis

Data management, analysis, and presentation were completed using the Statistical Package for the Social Sciences (SPSS) software, version 26.0 [33]. Descriptive statistics were used to produce the frequencies and percentages for all items in the questionnaire. Chi-square tests were used to find any correlations or significant differences between the categorical variables, where p was considered significant at *p* < 0.05.

## 3. Results

### 3.1. Survey Respondents’ Summary

Out of 691 approached students, 352 completed the questionnaire, thus giving a 51% response rate. In total, 48.5% (n = 178) of those studying in phase II of the undergraduate medical program responded, while 53.9% (n = 174) of those studying in phase III responded.

### 3.2. Descriptive Statistics

Table 3 shows the demographic and general characteristics of the study sample, which comprised 352 students aged 18–26 years, with a mean age of 22.1 years (±1.8 Standard Deviation). Most of the students (313 (88.9%)) were female. The sample included very similar numbers of phase II and phase III students (178 (50.6%) and 174 (49.9%), respectively). Regarding the computer literacy level, most of the students (265 (75.3%)) were competent. Around 206 students (58.5%) always used computer technology for learning at medical school, whereas 11 students (3.1%) never used it.

### 3.3. Students’ Perceptions towards AI

Table 4 shows the students’ awareness and perceptions of AI. The majority of students either strongly agreed or just agreed that AI will play an important role in healthcare (199 (56.5%) and 150 (42.6%), respectively). More than half of the students (240 (68.2%)) agreed that AI would replace some healthcare specialties during their lifetimes, while 112 students (31.8%) disagreed with it. When asked about their understanding of the basic computational principles of AI, 213 students (60.5%) reported that they understood them, whereas 139 students (39.5%) disagreed with it. Most of the students (329 (93.4%)) showed comfortability with terminologies related to AI.

The results showed that 238 students (67.4%) understood AI limitations, while 114 students (32.6%) did not. The majority of the students (329 (93.4%)) agreed that learning about AI would benefit their careers, and 289 students (82.1%) believed that all medical students should receive AI teaching or training.

Less than half of the students (144 (40.9%)) disagreed with the statement that they will be confident using AI tools at the end of their medical degrees; similarly, 159 students (40.2%) disagreed with the statement that they will have a better understanding of the methods used to assess the performance of healthcare AI at the end of their medical degrees. In addition, 145 students (41.1%) did not think that at the end of medical school, they would possess the knowledge needed to work with AI in routine clinical practice.

### 3.4. Student’s Perspective on the Impact of AI on Medical Education and Their Willingness to Use It

Table 5 highlights the impact of AI on medical education as seen by the students and their willingness to use it. The majority agreed that AI systems could have a positive impact on medical education and that incorporating AI into medical education would ease the learning process and prepare students for real clinical practice (233 (66.2%), 203 (57.7%), and 182 (51.7%), respectively). Most of the students (277 (78.7%)) did not think that AI would replace the roles of physicians in the future, and the willingness among the students to use AI in their medical education was high (322 (91.5%)).

### 3.5. Students’ Status for Previous Teaching or Training in AI

**Figure 1:**
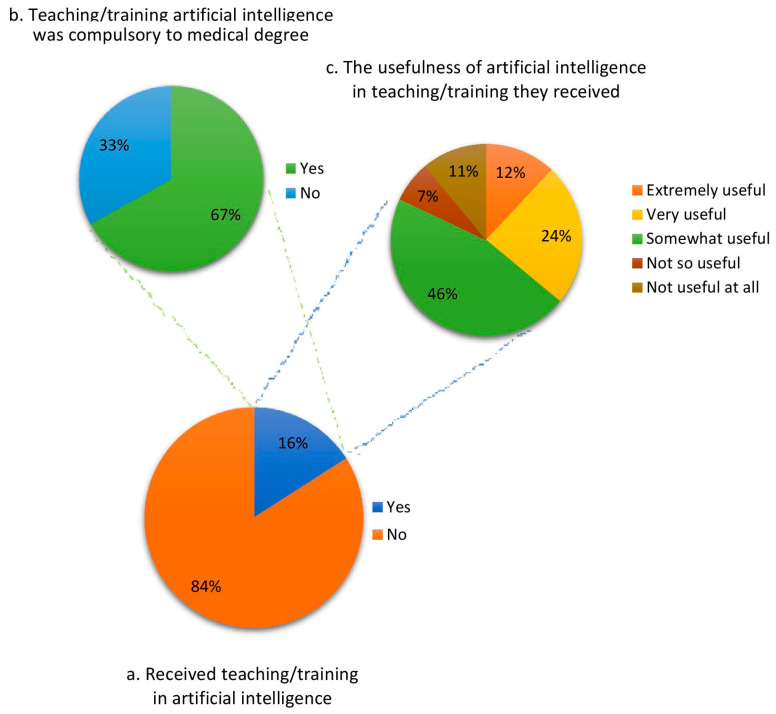
Previous teaching or training in artificial intelligence status of the study participants.

Figure 1 illustrates the status of the students in terms of whether they had received any teaching or training in AI. The majority of the students (296 (84%)) had not received any teaching or training in AI, while 56 students (16%) had earlier. Of those students who had received it, teaching or training was a compulsory part of the medical degrees for 38 students (67%), and 26 (46%) of them found it only somewhat useful. Moreover, seven students (12%) had found this teaching or training extremely useful, whereas six students (11%) had not found it useful at all.

### 3.6. The Association between Academic Years and the Perceptions towards AI

**Figure 2:**
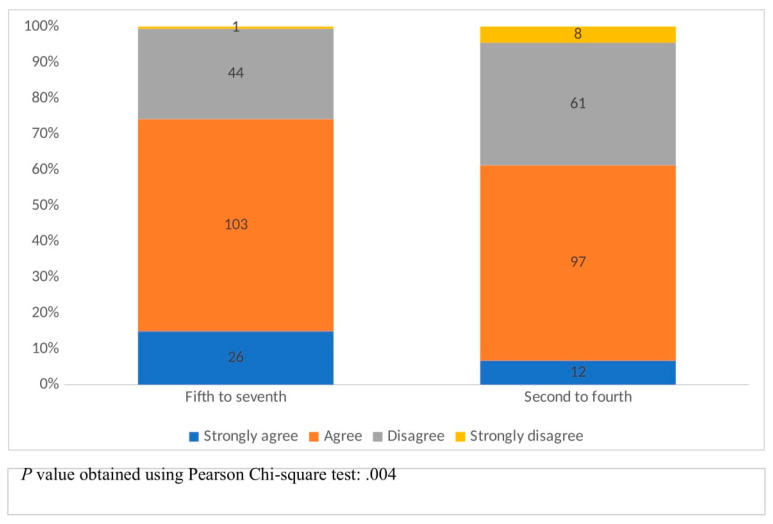
Students’ perceptions of an understanding of artificial intelligence limitations, according to the academic year.

Figure 2 represents students’ understanding of AI limitations according to their academic years. The four colors represent the levels of students’ agreement with the phrase “I understand artificial intelligence limitations”. The bar on the left portrays fifth to seventh-year (phase III) students, whereas the bar on the right portrays second to fourth-year (phase II) students. A Chi-square test for the association between academic year and agreement level generated a *p*-value of 0.004, with phase III students being more likely to agree with the phrase.

**Figure 3:**
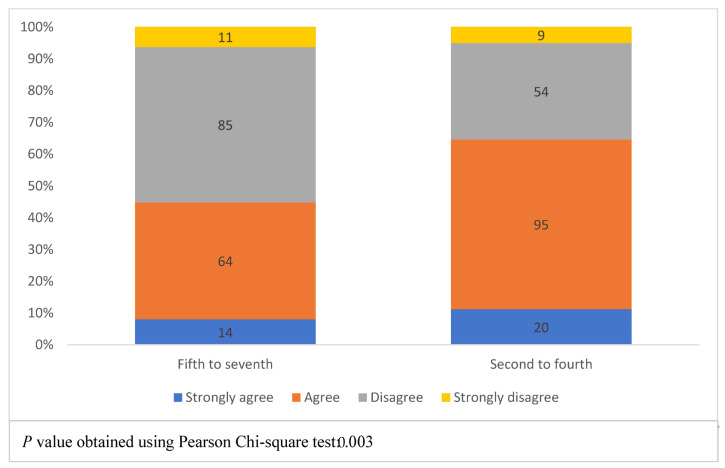
Students’ future expectation of better-understanding healthcare artificial intelligence performance, according to the academic year.

Figure 3 shows students’ future expectations of better-understanding healthcare AI performance, according to the academic years. The four colors represent the levels of students’ agreement with the phrase, “I will have a better understanding of the methods used to assess healthcare AI performance at the end of my medical degree”. The bar on the left shows fifth to seventh-year (phase III) students, while the bar on the right shows second to fourth-year (phase II) students. A Chi-square test for the association between academic year and agreement level resulted in a *p*-value of 0.003, with phase II students being more likely to agree with the statement.

**Figure 4:**
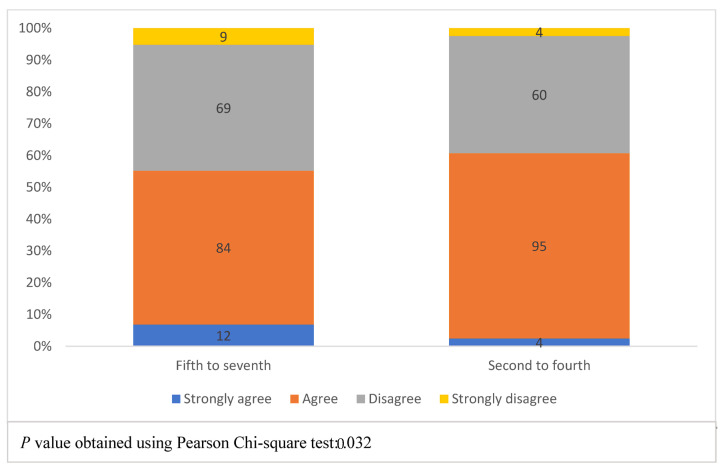
Students’ expectation of possessing the knowledge to work with artificial intelligence in practice, according to the academic year.

Figure 4 presents students’ expectations for possessing the knowledge required to work with AI in practice, according to the academic year. The four colors represent the levels of students’ agreement with the statement, “Overall, at the end of my medical degree, I feel I will possess the knowledge needed to work with AI in routine clinical practice”. The left bar shows the agreement levels of fifth to seventh-year (phase III) students, while the right bar represents second to fourth-year (phase II) students. A Chi-square test for the association between academic years and agreement level gave a *p*-value of 0.032, with phase II students being more likely to agree with the phrase.

### 3.7. The Association between Academic Years and Previous AI Teaching or Training, the Impact of AI on Medical Education and Willingness to Use It

With regards to the association between academic phases and previous AI teaching or training, Pearson’s Chi-square test found no significant association (*p* = 0.959). On the other hand, a significant association was found between the academic phase and believing that “AI will replace the roles of physicians in the future”, as more phase III students strongly disagreed with this statement compared to phase II students (*p* = 0.027). The results show that there was no significant association (*p* = 0.334) between academic phases and willingness to use AI in medical education.

## 4. Discussion

The mean age (22.1 ± 1.8) and male-to-female distribution (39 (11.1%) to 313 (88.9%)) of the sample reflected the characteristics of KUFoM’s students’ population of young ages and female predominance. Most of the students had competent computer skills and always used computer technology in their learning, as expected nowadays. The findings show that most of the students had positive perceptions of AI, and the majority were willing to adopt it in medical education.

### 4.1. Perceptions towards AI among Medical Students

AI has gained significant attention in healthcare lately and is proving to be an important aspect of the future of healthcare, including applications in various fields, such as pharmaceuticals, health informatics, image and scan analysis and medical devices [34]. The vast majority of the students believed that AI would play an important role in healthcare. Similar findings were reported for medical students in a United Kingdom (UK) study and in a United States of America (USA) study, in which more than 75% of the medical students believed that AI would have a moderate-to-major effect on medicine during their careers [5,35]. In our current study, it was not surprising to find that most of the students (93.4%) thought that receiving AI teaching would benefit their careers. This finding was consistent with other previous studies [4,5]. However, in the literature, examples of AI technology failures exist, such as IBM’s Watson for Oncology—an AI-assisted decision support system, which was adopted by a few hospitals but was later recalled for producing suboptimal results after millions of dollars had been spent on the project [36]. Moreover, experts have reported that AI and medical professionals complement each other, and although AI will change medical practice, it is unlikely to replace humans anytime soon—if ever [37,38]. In this current study, around two-thirds of the students believed that AI would replace some specialties in healthcare during their lifetimes, and similar findings were reported among almost half of the UK medical students in another study [5]. By contrast, the vast majority of the students (96.6%) in a study of German universities disagreed that physicians could be replaced by AI in the foreseeable future [4]. It is important to note that there could be a possibility that AI could be a substitute for human physicians for different activities in their practices, but this kind of replacement will not be absolute, as the AI capabilities will only augment the support of physicians for patient care and management; moreover, with the evolution of AI, clearer guidelines will emerge for its integration in the physicians’ practices and patients’ pathways [39].

In our study, the students generally reported that they understood AI terminology, limitations, and principles. Similar findings were found in a UK study, wherein the medical students said that they understood the basic computational principles associated with AI and its limitations but were not comfortable with AI terminology [5]. In a Canadian study, most of the medical students (78.9%) self-reported that they had a good understanding of AI; however, the same study used an assessment with true/false questions that included facts and fallacies about AI to objectively measure the students’ understanding of AI and concluded that a noticeably lower percentage actually understood it [40]. More than half of the students in our present study believed that at the end of their medical degrees, they would be able to use AI tools, apply AI in routine clinical practices, and assess the performance of healthcare AI. A possible explanation for these responses was that the medical students at Kuwait University possibly possessed an oversimplified understanding of AI, but the solidness of this understanding was not objectively assessed in this study, which was one of our study’s limitations. This is reinforced by the findings that only a small percentage of the sample had acquired their AI knowledge through training courses. In a cohort of UK medical students, the responses were drastically different, with the majority not feeling that they will be ready to work with AI, be confident in using AI tools if required, or understand the methods used to assess AI performance by the end of their degrees [5].

### 4.2. Teaching or Training in AI among Medical Students

The findings of our study reveal that a small percentage (a quarter) of the students had received some form of AI teaching or training, and no association was found between current academic phases (phase II vs. phase III) and previous AI teaching or training. The most likely reason for the majority of students not receiving AI teaching or training could be that AI courses are scarce and rarely offered at the faculty of medicine at KUFoM, which leaves only a small chance for students to receive such education or training as part of their program or even as extra-curricular work. This indicated a lack of a formal source of AI tuition in the academic institution, thus highlighting the need to incorporate AI concepts into the medical curriculum. It seemed that the vast majority of the students could have gained their AI knowledge from a myriad of sources that were not necessarily accurate or consistent. Previous studies from USA, Germany, Canada, and UK reported that the percentages of students who had been exposed to AI in an academic setting were 18%, 55.9%, 46.5%, and 9.2%, respectively. However, the nature of the exposure or teaching in each case was different. These studies found that the students who had received teaching or training in AI were more confident in their responses regarding understanding AI principles and showed greater readiness to use AI in clinical practices [4,5,35,40].

Our current study found that the majority of the students were willing to receive AI teaching or training as part of the medical program, which was similarly reported by students in the two studies of the UK and Germany [5,40]. Therefore, curriculum makers at medical schools need to act proactively and prepare students for future medical practices [24,41]. For instance, the University of Ulsan and Yonsei University in Korea offer AI-focused elective courses to their students [36]. Moreover, some universities have taken the initiative to apply the concepts of AI in medical education, in which medical students work in groups to create ideas-based technology for enhancing healthcare, such as at the Duke Institute for Health Innovation and the University of Virginia [24].

Incorporating computer-assisted learning or AI systems into the medical curriculum could supplement the conventional methods in several ways [22], namely: (1) using AI simulation systems for pre-clinical, clinical, and post-clinical learners to practice problem-based scenarios at different levels, as well as operate surgeries; and (2) using AI systems for performing artificial clinical examinations.

### 4.3. The Perceived Impact of AI on Medical Education and Willingness to Use It

Certainly, technology can transform people’s lives, and people interact with technology within an environment. A belief in the potential for AI applications to reform medical education was obvious among the students. Examples of various methods through which AI could transform the learning environments include intelligent tutoring systems that help to spot gaps in students’ knowledge and address them, virtual facilitators, data mining, and intelligent feedback [41]. In our study findings, the vast majority of students expressed positive attitudes toward the impact of AI in medical education and believed that it would make learning easier. Besides the fact that the current generation of students loves to use technology, it seems that the recent COVID-19 pandemic, with its substantial negative impact on medical education all over the world in general and in Kuwait specifically, could have influenced the students’ responses, tipping the scale even more towards favoring the use of AI in medical education. Moreover, most of the students believed that the use of AI in their medical education could prepare them for real clinical practices. This indicated that students perceived the importance and impact of AI systems as a supportive tool in the learning process. This was reinforced by the optimistic views of the students, as the vast majority of them were willing to use AI in their medical education.

### 4.4. Attitudinal Differences among the Students by Academic Phases

The study findings reveal that the phase III students believed they had a greater understanding of AI’s limitations compared to their phase II counterparts, but they showed less confidence in their responses in their ability to assess healthcare AI performance in the future or in possessing the knowledge required to work with AI in routine clinical practices at the end of their medical degrees. This could be owing to the fact that compared to their younger phase II colleagues, phase III students were currently in the realm of clinical training. Thus, they could have had a better awareness of the challenges of using AI in clinical practices. On the other hand, the phase II students had not yet experienced the clinical nature of medicine and would have their own (mostly) theoretical views on it later. In addition, the phase III students strongly disagreed with the statement that AI will replace their roles as physicians in the future. Similarly, this was expected from this group of students, who have seen closely the irreplaceable roles that physicians play in the various aspects of medical practices.

### 4.5. Risks and Ethical/Social Aspects of AI in Healthcare

Similar to other health information technologies, along with the potential benefits of AI and ML, pose possible risks that must be taken into consideration in medical practice; therefore, proper planning, implementation and governance are required to manage these risks [42]. Some of the risks/implications of AI and ML in clinical practices include human surveillance, medication errors, patient harm, patient safety, health inequalities, lack of transparency, mistrust, social biases, and vulnerabilities and breaches of data confidentiality/privacy [43,44,45]. Various mitigation measures and policy options could be implemented to minimize these risks of medical AI, such as multi-stakeholder engagement; increased transparency and traceability; clinical validation of AI apps and systems; and, finally, AI training and education for all stakeholders, including students, clinicians and patients [44]. In order to maximize the benefits of AI in medical practices from the educational perspective, sound knowledge of the various risks of AI, as well as ethical and social issues, needs to be inculcated in medical students as a part of their medical education before they utilize these technologies and start harnessing them in their future medical career.

### 4.6. The Implications of the Study Findings

The current study supports the use of AI in medical education, especially since the majority of medical students showed positive perceptions and attitudes towards AI in healthcare and medical education. Based on the findings of this study, there are several important implications that should be taken into account for adopting AI in medical education. Since a significant proportion of medical students believe that learning about AI would benefit their careers, hence, offering AI teaching or training for students would be of paramount importance. This could be achieved through an introductory course offered for students, as elective or mandatory, and including topics such as the basic principles of AI, big data analytics, applications of AI in medicine, ethical considerations, limitations, and future implications. Additionally, AI concepts can be integrated across existing courses, such as radiology, pathology, and diagnostic techniques. Workshops can be arranged for students where AI experts (data scientists or data engineers) are available to help students identify AI concepts in a practical way, wherein hands-on experience with AI applications can be acquired. The content and development of these courses and workshops should best align with the International Medical Informatics Association’s recommendations [46].

Moreover, it is encouraged to incorporate AI-based applications to help tutors in teaching conventional medical sciences. This can be applied by using the following: (a) AI-based simulators or virtual patient platforms to enhance the teaching of the conventional medical sciences, providing realistic scenarios for students to practice clinical decision-making skills and receive feedback in a safe environment; (b) Exploiting AI to conduct research projects in healthcare; (c) Technology-based AI systems, such as virtual reality or augmented reality simulations, incorporated into clinical training.

In addition, developing certification procedures for AI skills and competencies should be an objective of educational bodies [46]. Another valuable suggestion is post-graduate or parallel teaching or training in AI for medical graduates or students. Pathways include master and doctoral programs, fellowship-style training, and practical training courses [46].

### 4.7. Limitations

This study had several limitations. First, it was a single-center study. Second, based on the characteristics of the students who were willing to participate in the study, there might have been a possibility of selection bias. Third, the survey was based on a self-reported questionnaire that might have suffered from reporting bias, so the students might have exaggerated their responses with respect to their understanding of AI and their expected readiness to use it at the end of the medical program. Thus, it’s recommended for future studies to include an objective assessment as well, such as an assessment with true/false questions. Fourth, due to the lack of research on similar populations in the region, most of the results were compared with studies on related topics, but other populations could have different cultures and learning environments. Fifth, this study did not investigate why some students did not receive AI teaching or training, which might consist of interesting for future research. Finally, measuring the validity and reliability of the questionnaire with a larger sample of students is suggested for future studies, as the current study had a limited sample size. Moreover, it is recommended to consider multiple medical schools in future studies, as this would improve the generalizability of the results.

## 5. Conclusions

It can be concluded from the findings of this study that KUFoM students have positive perceptions towards AI systems, showing optimism towards learning more about AI in their medical education. Therefore, we recommended to the curriculum makers at KUFoM that AI teaching or training should be embedded into KUFoM’s undergraduate medical program. This could include AI principles, data science, and related ethical and legal issues. In addition, they also need to consider including the application of AI as part of medical education along with other advancements and innovations in medical practices. Undoubtedly, the anticipated role of AI in the future of medical practices and medical education is significant; however, paving the road for AI-dominated medical practice is required. Sound knowledge of the various risks of AI, as well as the ethical and social issues, needs to be inculcated in medical students as part of their medical education before they step into medical practice and start harnessing AI. It is vital to understand that AI is not here to replace doctors but will create new roles and opportunities for them in order to support their practice. The evolution and advancements of AI in the future will further clarify its potential directions of actual usage in the healthcare domain, along with its challenges and limitations.

## Figures and Tables

**Table 1:** Factor analysis of the “students’ perceptions” scale.

Items	Common Factor = 32.76% of Variance Loading
1. Artificial intelligence (AI) will play an important role in health care	0.482
2. AI will replace some specialties in healthcare during my lifetime	0.255
3. I understand basic AI principles	0.699
4. I am comfort with AI terminologies	0.670
5. I understand AI limitations	0.437
6. AI teaching will benefit my career	0.525
7. All medical students should receive AI teaching	0.472
8. I will be confident using AI tools at the end of my medical degree	0.691
9. I will have better understanding of the methods used to assess healthcare AI performance at the end of my medical degree	0.701
10. I will possess the knowledge needed to work with AI in routine clinical practice at the end of my medical degree	0.619
Eigen Value = 3.276	

**Table 2:** Factor analysis of the “Impact of artificial intelligence on medical education” scale.

Item No.	Common Factor = 46.64% of Variance Loading
1. AI systems would have a positive impact on medical education	0.811
2. Incorporating AI systems in medical education would ease your learning process	0.810
3. Use AI systems in medical education would prepare you for real clinical practice	0.750
4. Use AI systems in medical practice would replace your future role as a physician	0.176
5. The willingness of using AI in medical education system	0.651
Eigen Value= 2.332	

**Table 3:** Characteristics of the study population (n = 352).

Characteristic	n (%)
Age (in years)	
Mean ± SD	22.1 ± 1.8
Gender	
Male	39 (11.1)
Female	313 (88.9)
Current academic study year	
Second to fourth (Phase II)	178 (50.6)
Fifth to seventh (Phase III)	174 (49.4)
Computer literacy level	
Literate	66 (18.8)
Competent	265 (75.3)
Proficient	21 (5.9)
Usage of computer technology for learning	
Always	206 (58.5)
Sometimes	135 (38.4)
Never	11 (3.1)

**Table 4:** Perceptions towards artificial intelligence (n = 352).

Statement	Strongly Agree n (%)	Agree n (%)	Disagree n (%)	Strongly Disagree n (%)
AI will play important role in healthcare	199 (56.5)	150 (42.6)	2 (0.6)	1 (0.3)
AI will replace some specialties in healthcare during my lifetime	63 (17.9)	177 (50.3)	100 (28.4)	12 (3.4)
I understand basic AI principles	24 (6.8)	189 (53.7)	124 (35.2)	15 (4.3)
I am comfortable with AI terminologies	105 (29.8)	224 (63.6)	22 (6.3)	1 (0.3)
I understand AI limitations	38 (10.8)	200 (56.8)	105 (29.8)	9 (2.6)
AI teaching will benefit my career	105 (29.8)	224 (63.6)	22 (6.3)	1 (0.3)
All medical students should receive AI teaching	111 (31.5)	178 (50.6)	63 (17.9)	0
I will be confident using AI tools at the end of my medical degree	52 (14.8)	156 (44.3)	129 (36.6)	15 (4.3)
I will have better understanding of the methods used to assess healthcare AI performance at the end of my medical degree	34 (9.7)	159 (45.2)	139 (39.5)	20 (5.7)
I will possess the knowledge needed to work with AI in routine clinical practice at the end of my medical degree	28 (8)	179 (50.9)	129 (36.6)	16 (4.5)

**Table 5:** Impact of artificial intelligence on medical education and willingness to use it (n = 352).

Statement	Strongly Agree n (%)	Agree n (%)	Disagree n (%)	Strongly Disagree n (%)
AI systems will have a positive impact on medical education	107 (30.4)	233 (66.2)	12 (3.4)	0
Incorporating AI in medical education would ease the learning process	122 (34.7)	203 (57.7)	23 (6.5)	4 (1.1)
Using AI in medical education will prepare me for real clinical practice	94 (26.7)	182 (51.7)	70 (19.9)	6 (1.7)
AI will replace my future role as a physician	17 (4.8)	58 (16.5)	177 (50.3)	100 (28.4)
	**Very willing** **n (%)**	**Willing** **n (%)**	**Not willing** **n (%)**	**Not at all willing** **n (%)**
Willingness to use AI in medical education	120 (34.1)	202 (57.4)	27 (7.7)	3 (0.9)

## Data Availability

The datasets generated and/or analyzed in the current study are available from the corresponding author upon request. The data are not publicly available due to privacy.
